# Sex-divergent intrinsic brain function in Parkinson’s disease: elevated nigral fluctuations and premotor-visuospatial coupling in female patients

**DOI:** 10.3389/fnins.2026.1734858

**Published:** 2026-06-15

**Authors:** Ying Li, Lei Li, Xiuqin Jia, Lei Xu, Peipeng Liang

**Affiliations:** 1 Department of Radiology, Beijing Anzhen Hospital, Capital Medical University, Beijing, China; 2 Department of Encephalopathy II, Shanxi Acupuncture Hospital, Shanxi, China; 3 Department of Radiology, Beijing Chaoyang Hospital, Capital Medical University, Beijing, China; 4 School of Psychology and Beijing Key Laboratory of Learning and Cognition, Capital Normal University, Beijing, China

**Keywords:** amplitude of low-frequency fluctuations, fMRI, Parkinson’s disease, sex differences, substantia nigra

## Abstract

**Introduction:**

Cortical and subcortical alterations in brain intrinsic function have been widely reported in Parkinson’s disease (PD). However, sex differences in brain intrinsic function in PD are poorly understood. This study aimed to examine sex differences in spontaneous brain intrinsic function in PD and their associations with neuropsychological measurements.

**Methods:**

Using Parkinson’s Progression Markers Initiative (PPMI) resting-state fMRI, we compared amplitude of low-frequency fluctuations (ALFF) between male and female PD patients in substantia nigra (SN), globus pallidus (GP) and whole-brain. Subgroup analysis was performed, matching demographics with equal sample sizes. ALFF correlations with behavior were assessed by sex.

**Results:**

Female PD patients demonstrated higher ALFF in the right SN compared to males in the primary analysis. This sex difference achieved statistical significance in the demographics-matched subgroup analysis. Bilateral premotor ALFF was significantly elevated in female patients relative to males, independent of brain structure. Furthermore, right premotor ALFF showed a preliminary positive trend associated with visuospatial function exclusively in female PD patients.

**Conclusion:**

Female PD patients exhibit distinct functional signatures, primarily involving elevated premotor fluctuations and a preliminary premotor-visuospatial association. Preliminary SN alterations were also noted. These findings highlight the necessity of sex-stratified neuroimaging and provide preliminary support for premotor ALFF as a potential sex-divergent functional signature associated with cognitive profiling in female patients with PD.

## 1 Introduction

Parkinson’s disease (PD), characterized by progressive loss of dopaminergic neurons within the nigrostriatal pathway, leading to both motor and non-motor clinical manifestations (Lang and Lozano, 1998). This dopamine depletion triggers profound dysfunction within the cortico-basal ganglia-thalamo-cortical loops, which are essential for modulating motor control and cognitive processes (De Micco et al., 2019). Central to this pathophysiology are the substantia nigra (SN) and globus pallidus (GP), which serve as critical nodes in the “braking system” of the brain, regulating desired actions while inhibiting competing ones (Quartarone et al., 2020).

Epidemiological and clinical disparities between sexes are well-documented. Males exhibit a twofold higher incidence rate than females (Baldereschi et al., 2000), while females typically experience a later disease onset (Twelves et al., 2003). Beyond incidence, sex-related differences in phenotypic profiles are frequently observed; for instance, tremor tends to be more prevalent in females (Haaxma et al., 2007), whereas freezing of gait is more frequent in males (Kim et al., 2018). Furthermore, cognitive domains are differentially affected; while executive functions and global cognition are relatively preserved in females, they often exhibit more pronounced impairment in the visuospatial domain compared to males (Lin et al., 2018). Motor symptoms develop earlier in male patients and have faster progression (Haaxma et al., 2007). Furthermore, these sex-related disparities extend to clinical management and advanced interventions; for instance, recent evidence highlights a persistent “gender gap” in the utilization and outcomes of deep brain stimulation (Somma et al., 2024). These sex differences in clinical and cognitive profiles highlight the need for sex-divergent investigation of functional brain alterations in PD.

Despite these observed clinical and cognitive sex-related differences, the underlying functional brain mechanisms remain insufficiently understood. Current neuroimaging research into the sex-divergent neural patterns of PD has yielded inconsistent results, often characterized by small sample sizes or varied methodologies. To date, only a limited number of studies have explored this area; for instance, while some research reported increased sensorimotor network connectivity in males (De Micco et al., 2019), others found no significant sex-related differences in dynamic functional connectivity (Tang et al., 2017). Notably, most existing studies have focused on inter-regional coordination (connectivity), including the investigation of altered functional connectivity in deep subcortical nodes such as the dentate nucleus (Liu et al., 2013). This focus leaves a notable gap in our understanding of how spontaneous regional brain activity inherently fluctuates in male versus female PD patients. Given that regional functional alterations often precede large-scale network disruptions (Bullmore and Sporns, 2009), investigating these localized BOLD fluctuations is essential to provide a more granular perspective on the functional signatures associated with sex-related clinical phenotypes in PD.

To address this gap, analysis of blood-oxygen-level-dependent (BOLD) signal dynamics offers a valuable approach for characterizing intrinsic brain activity patterns. Specifically, the Amplitude of Low-Frequency Fluctuation (ALFF) serves as a sensitive metric for quantifying the magnitude of local spontaneous BOLD fluctuations. While ALFF is widely utilized as a proxy for regional functional integrity, it provides a unique perspective on the physiological signatures of brain regions involved in PD pathology, independent of inter-regional connectivity (Wang et al., 2025; Zang et al., 2007). Distinct from connectivity-based measures that focus on inter-regional coordination, ALFF enables a localized assessment of functional fluctuations. This makes it particularly suitable for identifying regional alterations within the basal ganglia and cortex—areas frequently associated with the clinical presentations observed in male and female PD patients. Furthermore, as regional functional signals in neurodegenerative contexts can be significantly impacted by structural variations, it is essential to account for underlying morphological changes. Therefore, we integrated regional gray matter measures (e.g., density or volume) as covariates in our statistical models to ensure that the observed functional variations reflect more than just the secondary effects of local atrophy. This multi-modal consideration aims to provide a more refined characterization of the sex-related functional patterns in PD.

In the present study, we aimed to explore sex-divergent patterns in brain structure and regional BOLD fluctuations among patients with PD. To provide a comprehensive assessment, we first performed a region-of-interest (ROI) analysis focused on the bilateral SN and GP, given their pivotal roles in PD pathology, followed by a voxel-wise whole-brain exploratory analysis. Additionally, we investigated the potential associations between these regional brain alterations and neuropsychological measurements. This investigation aims to provide insights into the sex-divergent patterns of intrinsic brain function in PD, potentially informing the future development of personalized, sex-sensitive clinical management strategies within the field.

## 2 Materials and methods

### 2.1 Subjects

This study utilized structural and resting-state functional magnetic resonance imaging (rs-fMRI) data from the Parkinson’s Progression Markers Initiative (PPMI)^1^ cohort (Marek et al., 2018), with all data collected up to December 1, 2020. This analysis included 96 individuals diagnosed with PD (65 males) and 22 controls (17 males), all of whom completed T1-weighted and rs-fMRI scans. PD diagnosis required ≥ 2 cardinal motor symptoms (bradykinesia, rigidity, or resting tremor) with asymmetric presentation, plus dopamine transporter deficit on imaging. Exclusion criteria comprised a history of significant neurological, psychiatric, or nasal disorders, as well as use of medications that could potentially affect dopamine transporter SPECT results. All PPMI sites obtained Institutional Review Board approval, with participants providing written informed consent prior to enrollment.

### 2.2 Clinical and neuropsychological assessment

Motor severity was quantified using the Movement Disorder Society-Unified Parkinson’s Disease Rating Scale Part III (MDS-UPDRS III) and Hoehn and Yahr (H&Y) staging. Global cognition was measured with the Montreal Cognitive Assessment (MoCA), while domain-specific functions were measured by: Hopkins Verbal Learning Test (HVLT; verbal memory), Judgment of Line Orientation (JoLO; visuospatial function), Letter-Number Sequencing/Semantic Fluency Test (LNS/SFT; executive function), and Symbol Digit Modalities Test (SDMT; attention). Depression and anxiety were evaluated using the Geriatric Depression Scale (GDS) and State-Trait Anxiety Inventory (STAI), respectively. All demographic and clinical data appear in Table 1.

**TABLE 1:** Demographic and neuropsychological characteristics.

Characteristics	FPD (*n* = 29)	MPD (*n* = 53)	NC (*n* = 17)	ANOVA/*t*-test	*Post-hoc*		
				P	P1	P2	P3
					**(NC vs. MPD)**	**(NC vs. FPD)**	**(MPD vs. FPD)**
Age (years)	59.62 ± 9.89	62.26 ± 10.57	59.35 ± 11.59	0.405	0.942	1.000	0.795
Education (years)	15.62 ± 3.29	15.37 ± 2.76	16.89 ± 2.50	0.159	0.168	0.460	1.000
Disease duration (months)	6.93 ± 9.33	6.23 ± 6.77	—	0.683	—	—	—
H-Y Stage	1.73 ± 0.46	1.71 ± 0.46	—	0.871			
MDS-UPDRS III	20.69 ± 10.03	23.94 ± 10.78	—	0.171	—	—	—
GDS	2.51 ± 2.91	2.45 ± 2.73	1.00 ± 1.65	0.114	0.143	0.191	1.000
STAI	67.56 ± 19.21	65.65 ± 17.99	61.88 ± 15.76	0.589	1.000	0.915	1.000
MoCA	27.55 ± 2.54	26.94 ± 2.32	28.12 ± 1.05	0.121	0.168	1.000	0.670
Visuospatial							
JoLO	10.68 ± 2.88	12.72 ± 2.41	14.00 ± 2.06	**< 0.001**	0.187	**< 0.001**	**0.001**
Memory							
HVLT-R recall	48.55 ± 12.99	45.48 ± 12.74	46.00 ± 12.87	0.559	1.000	1.000	0.856
HVLT-R recognition	47.52 ± 12.05	45.20 ± 12.71	39.47 ± 16.19	0.134	0.336	0.142	1.000
Executive							
LNS	11.59 ± 2.61	11.15 ± 2.48	11.35 ± 2.40	0.738	1.000	1.000	1.000
SFT	52.51 ± 14.69	53.31 ± 10.23	49.41 ± 10.06	0.466	0.652	1.000	1.000
Attention							
SDMT	46.27 ± 9.45	43.18 ± 10.30	49.47 ± 9.86	0.054	0.069	0.893	0.512

Data are expressed as the mean ± standard deviation. *P*-values were derived from one-way ANOVA and two-sample *t*-tests for parametric tests. MDS-UPDRS III, Movement Disorder Society-Unified Parkinson’s Disease Rating Scale; H-Y, Hoehn and Yahr; GDS, Geriatric Depression Scale; JoLO, Judgment of Line Orientation; HVLT-R, Hopkins Verbal Learning Test-Revised; LNS, Letter-Number Sequencing; SFT, Semantic Fluency Test; STAI, State-Trait Anxiety Index; MoCA, Montreal Cognitive Assessment; SDMT, Symbol-Digit Modalities Test; NC, normal control; PD, Parkinson’s disease. FPD, female Parkinson’s disease patients; MPD, male Parkinson’s disease patients. Bold values indicate statistically significant differences (*p* < 0.05).

### 2.3 MRI data acquisition

Structural imaging utilized a T1-weighted 3D magnetization-prepared rapid gradient echo (MP-RAGE) sequence with repetition time (TR) = 2,300 ms, echo time (TE) = 3.0 ms, flip angle = 9°, matrix size = 256 × 240, and 176 sagittal slices at 1 mm thickness. Rs-fMRI employed echo-planar imaging (EPI) acquiring 210 volumes (TR = 2,400 ms, TE = 25 ms, flip angle = 80°, field of view = 224.4 × 217.8 mm^2^, matrix = 68 × 66 (reconstructed from a 476 × 462 mosaic), slice thickness = 3.3 mm). Clinical assessments were synchronized with rs-fMRI acquisition timing.

### 2.4 MRI data preprocessing

Data preprocessing utilized SPM12 with Data Processing Assistant rs- fMRI (DPARSF) (Chao-Gan and Yu-Feng, 2010). Rs-fMRI processing included: discarding initial 10 timepoints, slice timing correction, realignment, MNI normalization, 6-mm FWHM smoothing, and nuisance regression [Friston 24-parameter model plus white matter (WM) and cerebrospinal fluid (CSF) signals via CompCor, and linear detrending]. ALFF was computed in 0.01–0.08 Hz band (Yang et al., 2007).

Structural analysis employed voxel-based morphometry (VBM): T1 images segmented into gray matter (GM), WM, and CSF probability maps within MNI space via the unified segmentation approach in SPM12, DARTEL-generated study-specific template (Ashburner, 2007). Then, all GM segments were nonlinearly normalized to this template, and modulation was applied using the Jacobian determinants to account for volumetric changes during spatial normalization (Good et al., 2001). The modulated GM maps (voxel size: 1.5 × 1.5 × 1.5 mmł) were subsequently smoothed. Total intracranial volume (TIV) summed tissue volumes.

Region-of-interest (ROI) analysis targeted bilateral SN and GP according to the Automated Anatomical Labeling Atlas 3 (AAL3) (Rolls et al., 2020), extracting ALFF and GM volume for statistics.

### 2.5 Quality control procedure

The quality control (QC) is checked in this study as follows. First, checking the number of volumes and reorienting the image to the template image in MNI space, the participants with fewer volumes and QC scores of images below 3 were excluded. QC scores are based on the DPABI software by visual assessment and manually orientation-adjusting the structural and functional images. Second, the subjects with a head-motion values larger than 2 times of the standard deviation were excluded. Third, visual assessment the co-registration between structural images and functional images and the spatial registrations of the fMRI images to an MNI space template excluded the subjects with bad co-registration, MRI artifacts and flipped image direction. Finally, to address motion- and physiology-derived global artifacts, time series were evaluated via global signal removal in the supplementary analysis. In addition, in the ROI analysis, outlier data, defined as values > 2 times the standard deviation, were excluded.

### 2.6 Statistical analysis

A one-way analysis of variance (ANOVA) was performed to evaluate differences across groups in demographic and clinical parameters (age, years of education, MoCA scores, and cognitive assessments) among normal controls (NC), male PD patients, and female PD patients. *Post-hoc* pairwise analyses were carried out using Bonferroni correction (SPSS 26.0). Independent *t*-tests further compared disease duration, H-Y stage, and MDS-UPDRS III scores between male and female PD participants (SPSS version 26.0).

To isolate functional alterations independent of structural changes, we included gray matter (GM) as a covariate in all statistical models. Specifically, voxel-wise GM density maps were used as covariates for the whole-brain ALFF analysis, while the mean GM volume of each corresponding ROI was included as a covariate for the ROI-based analyses. Prior to these analyses, a formal multicollinearity diagnostic was performed to ensure that the inclusion of GM metrics and clinical covariates (e.g., MDS-UPDRS III) did not bias the estimation of sex-related effects.

#### 2.6.1 Confirmatory ROI analysis

One-way analysis of covariance (ANCOVA) was conducted to compare ALFF and GM volume across each ROI. For comparisons between male and female PD patients, covariates included MDS-UPDRS III score, age, education, TIV (for GM volume), or GM volume (for ALFF). To control for multiple testing among the four pre-defined ROIs, a significance threshold of *p* < 0.013 (calculated as 0.05/4) was established using Bonferroni correction.

#### 2.6.2 Exploratory whole-brain analysis

General linear model (GLM)-based analyses were applied to ALFF and GM volume maps across the whole brain to identify sex-divergent patterns.

##### 2.6.2.1 Voxel-wise discovery

Sex differences in ALFF and GM volume within the PD group were tested using ANCOVAs. For GM volume, covariates included age, education, MDS-UPDRS III scores, and TIV. For ALFF, covariates included age, education, MDS-UPDRS III scores, and GM density to isolate functional alterations independent of structural atrophy. The significance threshold was set at voxel-level *p* < 0.001 and cluster-level *p* < 0.05 (Gaussian Random Field, GRF-corrected).

#### 2.6.2.2 *Post-hoc* characterization

For brain regions exhibiting significant sex differences in ALFF, mean values were extracted to further evaluate their disease-specificity. Specifically, these values were contrasted between the NC group and each PD subgroup (Female PD vs. NC and Male PD vs. NC) using ANCOVAs (adjusting for age, education, and TIV). To account for multiple testing across the n identified clusters, a Bonferroni-corrected threshold of *p* < 0.05/n was adopted.

#### 2.6.3 Multiple linear regression between ALFF values and neuropsychological assessments in the PD group

Multiple linear regression was performed with cognitive profiles demonstrating significant sex-based differences within the PD cohort as the dependent variable. The primary imaging predictors were defined as the ALFF values of regions that exhibited significant sex differences in either the whole-brain or ROI-based analyses. Age, education, and TIV were included as covariates to control for demographic and structural influences. Additionally, SDMT was included as a covariate to account for its potential confounding effects on information processing speed and attention. To address multiple comparisons among the identified imaging predictors, a Bonferroni-corrected threshold of *p* < 0.05/n was adopted, where n represents the number of regions with significant sex differences. Statistical analyses were conducted with IBM SPSS Statistics (version 26.0).

### 2.7 Supplementary analysis

Global signals are associated with head motion, cardiac rhythms, and respiration in addition to physiological neural information (Li et al., 2019). Methodologically, global signal regression (GSR) has been proven to be highly efficient at reducing global artifacts caused by motion and other physiological fluctuations. To ensure the reliability of the results, GSR in rs-fMRI data preprocessing was further performed.

Additionally, the total sample size analysis may suffer from statistical bias (Linden and Samuels, 2013). To eliminate potential bias from sample imbalances, a sensitivity analysis was performed in a strictly demographic-matched subset (29 female PD vs. 29 male PD). In this validation phase, we employed an extraction-based approach for all regions. Mean ALFF values from the four pre-defined ROIs and the n clusters identified in the whole-brain analysis were extracted and compared between sex-matched PD groups using ANCOVAs. Strict Bonferroni correction (*p* < 0.05/4 for ROIs and *p* < 0.05/n for whole-brain clusters) was applied to ensure the robustness of our conclusions. The spatial consistency of ALFF patterns with and without global signal regression (GSR) was confirmed (see Supplementary material part I).

## 3 Results

### 3.1 Participants and demographic characteristics

**FIGURE 1:**
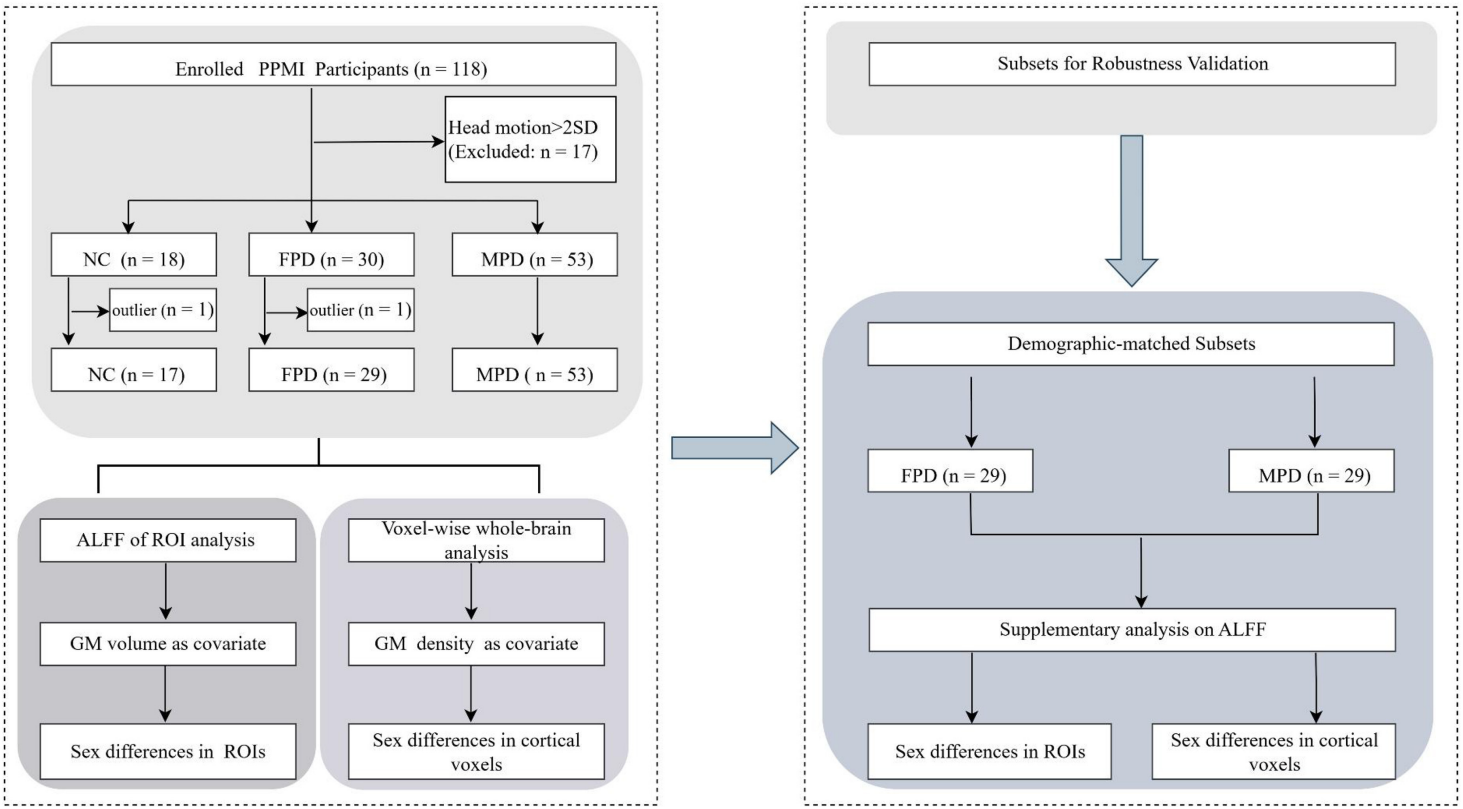
Study flowchart. The left panel illustrates the primary analysis workflow, detailing the exclusion criteria based on technical quality control (head motion > 2 SD) and statistical outlier removal. It also defines the structural covariate strategies for ROI-based and voxel-wise analyses. The right panel displays the validation framework using a demographically matched subset (*n* = 29 per group) to ensure the robustness of the findings. NC, healthy controls; FPD, female Parkinson’s disease; MPD, male Parkinson’s disease; ALFF, amplitude of low-frequency fluctuation; GMD, gray matter density; SD, standard deviation.

After conducting quality control, rs-fMRI data from 53 male PD patients, 29 female PD patients, and 17 NC were analyzed in this study (Figure 1).

Table 1 summarizes the between-group comparisons of demographic and neuropsychological characteristics. No significant between-group differences were observed in demographic (age, education) or psychological variables (GDS, STAI) across the three groups (*p* > 0.05). Notably, female PD patients exhibited significantly lower JoLO scores compared to males and NC, whereas other neuropsychological tests yielded no significant group differences (*p* > 0.05). Motor-related variables, including disease duration, Hoehn-Yahr stage, and MDS-UPDRS III scores, were also comparable between female and male PD patients (*p* > 0.05), as detailed in Table 1.

To evaluate the stability of our findings, a demographic-matched subset analysis was performed. Detailed results are provided in Supplementary material part I: Robustness Analyses.

### 3.2 Sex differences in ROIs analysis

**FIGURE 2:**
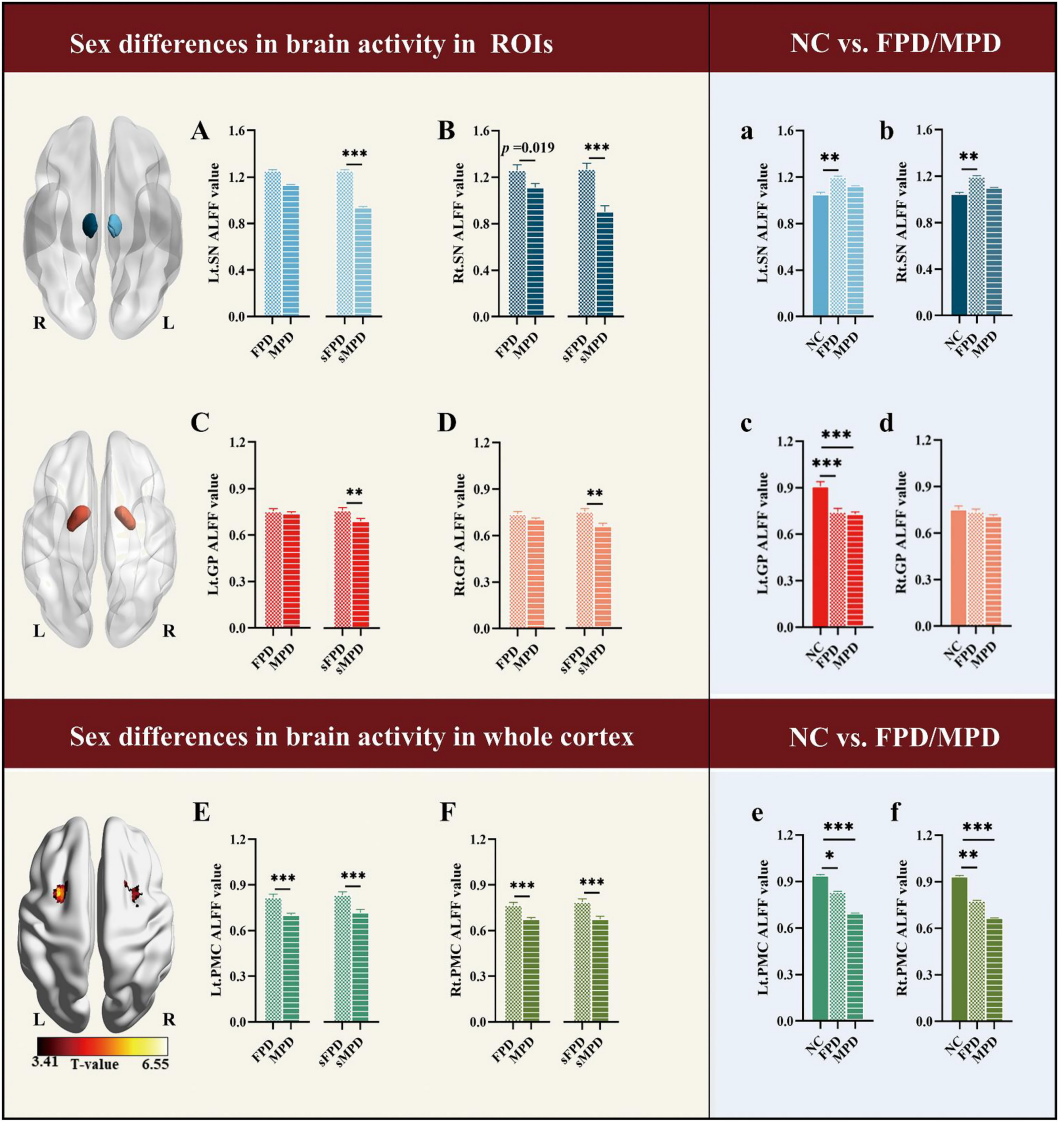
Sex disparities intrinsic regional fluctuations: Regional (ROIs) and whole-brain cortical findings. The upper panels show adjusted mean ALFF values in ROIs (bar charts) and their anatomical definitions (brain maps and histogram: blue = SN [dark = right, light = left], red = left GP, orange = right GP). Comparisons between FPD and MPD (adjusted for age, education, MDS-UPDRS III, and GM volume) revealed a trend toward higher right SN ALFF in FPD (*p* = 0.019), which was confirmed as a significant difference in subgroup analyses (*p* < 0.001) (shown in **B**). NC vs. PD comparisons (adjusted for age, education, and GM volume) showed higher bilateral SN ALFF in FPD vs. NC **(a,b)**, while both FPD and MPD had lower left GP ALFF than NC **(c)**. The lower panel highlights whole-cortex brain regions with sex differences in ALFF in PD patients. FPD exhibited higher bilateral PMC ALFF than MPD (GRF-corrected *p* < 0.001, cluster *p* < 0.05), confirmed in subgroup comparisons **(E,F)**. Both PD groups showed reduced PMC ALFF vs. NC (adjusted for age, education, and TIV; **e,f**). Lt., Left; Rt., Right; SN, Substantia nigra; GP, Globus pallidus; NC, Normal control; FPD, Female Parkinson’s disease patients; MPD, Male Parkinson’s disease patients; sFPD, FPD of the sample size matched group; sMPD, MPD of the sample size matched group; ROI, Region of interest; ALFF, Amplitude of low-frequency fluctuation; PMC, premotor cortex. ***p< 0.001; **p< 0.01; *p< 0.05. **(A,C,D,d)** Show the inter-group comparisons in the remaining regions of interest (ROIs), where no statistically significant differences were observed.

No significant sex-related differences were observed in GM volume across the four ROIs. However, in the primary analysis of the total sample, a trend toward sex differences in the right SN was observed (*p* = 0.019), though it did not survive the strict Bonferroni-corrected threshold (*p* < 0.013). However, in the subsequent Robustness analysis using the demographic-matched subset, this difference became statistically significant (*p* < 0.013, see Figure 2 in the main text and Supplementary material part I).

Compared to the NC, male PD patients demonstrated increased GM volume in bilateral GP, while female PD patients showed higher ALFF values in bilateral SN. Both PD groups displayed reduced ALFF in the left GP relative to NC (Figure 2 and Table 2).

**TABLE 2:** Comparisons of the VBM and ALFF alterations for ROIs.

	VBM			ALFF			
	P1	P2	P3	P1	P2	P3	P4
ROIs	(NC vs. MPD)	(NC vs. FPD)	(MPD vs. FPD)	(NC vs. MPD)	(NC vs. FPD)	(MPD vs. FPD)	(sMPD vs. sFPD)
Lt.SN	0.144	0.791	0.243	0.096	**0.007**	0.053	**0.000**
Rt.SN	0.120	0.173	0.382	0.048	**0.003**	**0.019**	**0.000**
Lt.GP	**0.004**	0.184	0.454	**0.000**	**0.000**	0.424	**0.008**
Rt.GP	**0.000**	0.136	0.142	0.260	0.574	0.072	**0.001**

VBM, voxel-based morphometry; ALFF, amplitude of low-frequency fluctuation; P4, *p*-values of comparison between MPD and FPD in the sample-matched group. Lt., left; Rt., right; SN, substantia nigra; GP, globus pallidus. NC, normal control; FPD, female Parkinson’s disease patients; MPD, male Parkinson’s disease patients; sFPD, female Parkinson’s disease patients in sample size matching group; sMPD, male Parkinson’s disease patients in sample size matching group. The Bonferroni corrected *p* level was 0.05/4 = 0.013. Bold values indicate statistically significant differences after Bonferroni correction (*p* < 0.05/4 = 0.0125); values showing a marginal trend toward significance such as *p* = 0.019) are also bolded for exploratory purposes.

### 3.3 Sex differences in the whole brain cortex analysis

A lower GM volume was observed in the bilateral fusiform gyrus (FG) (Brodmann area, BA 19/37) and brainstem in females relative to male PD patients (Supplementary Table S1). However, higher ALFF in the bilateral premotor cortex (PMC) (BA 6) was found in females than in male PD patients (Figure 2 and Supplementary Table 2). Furthermore, compared to NC, both PD groups had reduced PMC ALFF bilaterally (*p* < 0.025, Figure 2), while whole cortex for GM volume and ALFF differences between NC and PD subgroups are reported in Supplementary Tables 1, 2.

### 3.4 Multiple linear regression results

**FIGURE 3:**
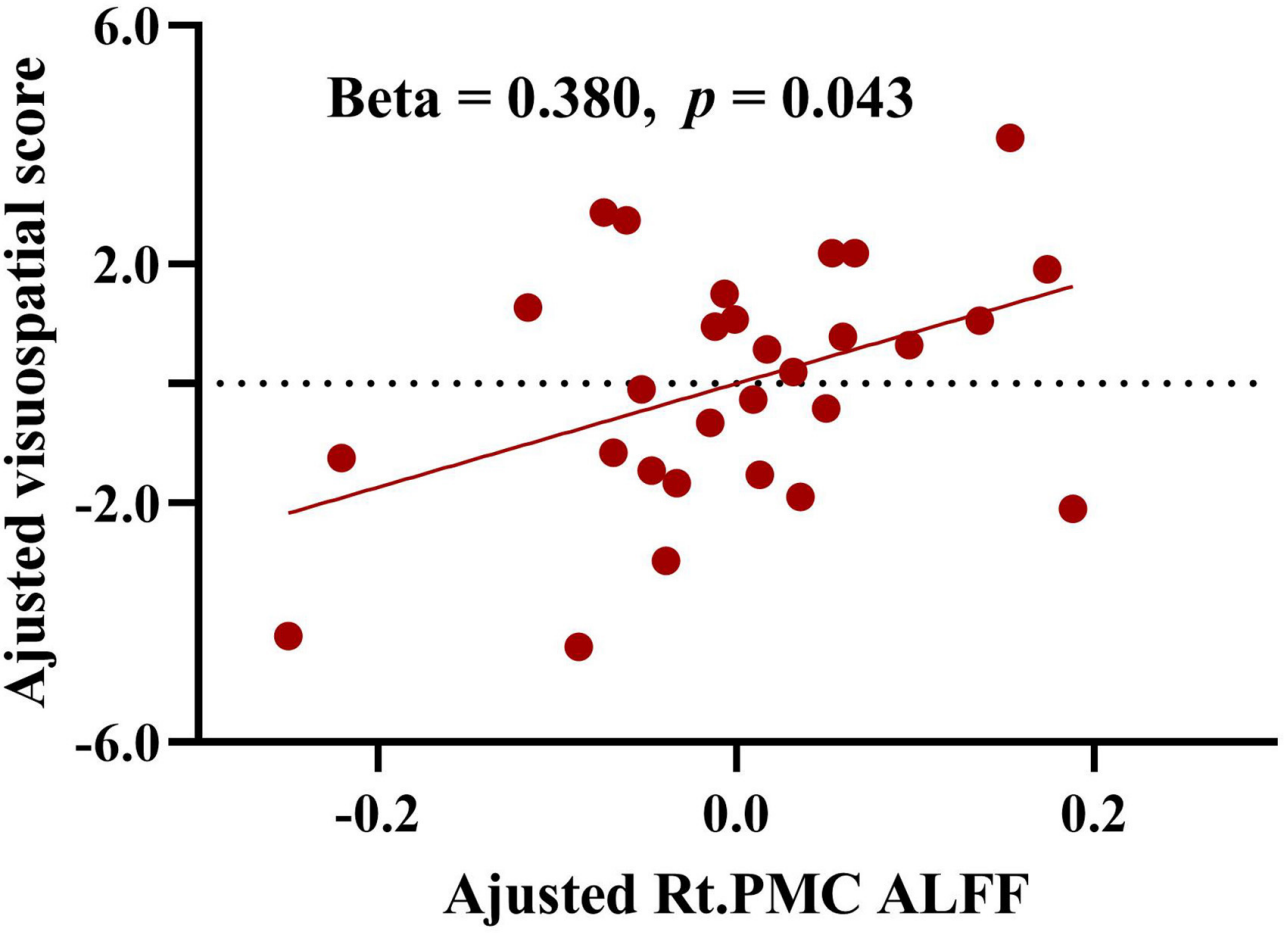
Partial regression plot of visuospatial score and ALFF in the right PMC in female PD patients. The scatter plot showing the association between visuospatial score and ALFF in the PMC after adjusting for age, education, TIV, SDMT, ALFF in left PMC, and ALFF in the right SN. Each point represents an individual patient. The solid line indicates the fitted regression line (slope β = 0.380, *p* = 0.043). PMC, premotor cortex; SN, substantia nigra; TIV, total intracranial volume; SDMT, Symbol Digit Modalities Test. Rt., Right; ALFF, Amplitude of low-frequency fluctuation; FPD, Female Parkinson’s disease patients.

In female and male PD patients, the visuospatial test score was set as the dependent variable (y), and age, education, TIV, SDMT (attention test), ALFF values of the right PMC, left PMC, and the right SN were set as the independent variables in female and male PD patients (Figure 3 and Table 3).

**TABLE 3:** Result of multiple linear regression with the visuospatial score as the dependent variable.

Group		Unstandardized coefficients		Standardized coefficients			
		*B*	Std. error	Beta	*t*	Sig.	VIF
FPD	(Constant)	–18.050	9.804		–1.841	0.080	
	Age (χ_1_)	0.020	0.045	0.067	0.440	0.665	1.167
	Education (χ_2_)	0.008	0.152	0.009	0.053	0.958	1.504
	TIV(χ_3_)	0.013	0.004	0.458	2.942	**0.008**	1.206
	SDMT (χ_4_)	0.158	0.050	0.520	3.184	**0.004**	1.327
	Rt.PMC ALFF(χ_5_)	8.677	4.025	0.380	2.156	**0.043**	1.551
	Lt.PMC ALFF(χ_6_)	–6.528	4.464	–0.271	–1.462	0.158	1.707
	Rt.SN ALFF(χ_7_)	0.380	1.569	0.038	0.242	0.811	1.224
ANOVA: *P* = 0.005; *R*^2^ = 0.58							
MPD	(Constant)	7.323	6.508		1.125	0.265	
	Age (χ_1_)	–0.050	0.029	–0.218	–1.692	0.096	1.054
	Education (χ_2_)	–0.046	0.123	–0.053	-0.374	0.709	1.256
	TIV(χ_3_)	0.004	0.003	0.215	1.598	0.116	1.145
	SDMT (χ_4_)	0.013	0.030	0.054	0.424	0.673	1.022
	Rt.PMC ALFF(χ_5_)	–2.917	5.456	–0.090	–0.535	0.595	1.777
	Lt.PMC ALFF(χ_6_)	3.598	3.960	0.138	0.909	0.367	1.468
	Rt.SN ALFF(χ_7_)	1.187	1.270	0.122	0.934	0.354	1.080
ANOVA: *P* = 0.503							

ALFF, amplitude of low-frequency fluctuation; TIV, total intracranial volume; SDMT, Symbol-Digit Modalities Test; PMC, premotor cortex; SN, substantia nigra; FPD, female Parkinson’s disease patients; MPD, male Parkinson’s disease patients; L, left; R, right; VIF, variance inflation factor. Bold values indicate statistically significant differences (*p* < 0.05).

In the female PD group, the multiple linear regression model significantly predicted visuospatial performance (*p* = 0.005, *R*^2^ = 0.58). Based on the preceding whole-brain and ROI analyses, three regions exhibiting sex differences (right PMC, left PMC, and right SN) were included as primary imaging predictors. Consequently, the Bonferroni-corrected threshold was set at *p* < 0.0167 (0.05/3). After correction, SDMT (Beta = 0.520, *p* = 0.004) and TIV (Beta = 0.458, *p* = 0.008) remained significant independent predictors. The right PMC ALFF exhibited a preliminary positive trend associated with visuospatial scores (Beta = 0.380, *p* = 0.043), although it did not survive the strict correction for the three imaging predictors. Furthermore, the variance inflation factor (VIF) was < 2, which suggests that there was no collinearity between variables. In addition, no relationship was found between visuospatial score and independent variables in male PD patients.

## 4 Discussion

This study conducted a comparison of sex differences in PD in terms of neuropsychology, brain structure and function. With regard to neuropsychological test, the result confirmed the previous evidence that visuospatial function was more impaired in females than males in PD patients (Haaxma et al., 2007; Lin et al., 2018). Thus, the sex-related differences in visuospatial function are consistent findings across studies.

Female PD patients exhibited higher ALFF values in the right SN compared to males. While the sex-related difference in right SN ALFF observed in the total sample did not survive strict multiple comparison correction, its emergence in the matched sub-sample suggests a potential functional shift that warrant further investigation. These divergent patterns of regional fluctuations might be associated with sex-related biological substrates, including variations in striatal binding (Chen et al., 2023; Haaxma et al., 2007), dopaminergic neuron density in the SN (Leranth et al., 2000), dimorphic gene expression profiles in the SN (Cantuti-Castelvetri et al., 2007). Previous studies ranging from animal models to human research have suggested potential female- divergent biological characteristics within neurological contexts (Seyfried et al., 2018; Tamás et al., 2005). A robust body of evidence underscores the neuroprotective capacity of estrogens and their sex-specific modulation of dopaminergic pathways (Gillies and McArthur, 2010). However, it remains unclear whether the higher ALFF observed in female PD patients directly reflects these biological factors or represents a distinct functional response to underlying pathology. Yet, in this work, female PD patients were shown higher intrinsic functional fluctuations in the bilateral SN than NC. The observed higher ALFF values in the bilateral SN of female PD patients relative to NC may represent a sex-related functional shift in response to nigral neurodegeneration. However, whether these altered fluctuations reflect a functional reorganization to maintain output or a pathological shift in spontaneous neural activity remains speculative. Our current resting-state design does not provide the metabolic or neurochemical evidence required to distinguish between these underlying mechanisms.

In contrast, relative to NC, the ALFF of the left GP was decreased in both PD groups in this study. This result is in line with the results of the proton MR spectroscopy study, suggesting that GP damage may occur earlier in the disease process and may progress faster than SN impairment (Wu et al., 2016). A meta-analysis confirmed this result (Gu et al., 2022).

For the whole brain cortex analysis, it was found that the ALFF values in the bilateral PMC were higher in female than male PD patients. To investigate whether this divergence reflects an innate biological sex difference, we performed a supplementary analysis using an independent cohort of healthy controls from the PPMI database (14 females, 10 males). The absence of significant ALFF sex differences in the PMC within this healthy cohort preliminarily suggests that the observed patterns in our study might be associated with PD-related pathology rather than innate physiological dimorphism. However, given the limited sample size of this supplementary cohort, these findings should be interpreted as supportive rather than definitive evidence of disease-specificity. Notably, we identified a preliminary positive trend between right PMC ALFF and visuospatial scores within the female PD group, an association that was not observed in the male PD group. Although this association did not survive the strict Bonferroni correction, the substantial effect size (Beta = 0.380) suggests that the PMC might act as a potential functional node in the sex-divergent manifestation of visuospatial dysfunction in PD. Given the subgroup sample size, this hypothesis-driven regression should be viewed as exploratory. Potential overfitting remains a concern, and these findings require validation in larger independent cohorts.

While both PD subgroups exhibited lower bilateral PMC ALFF relative to NC—reflecting a general disease-related reduction in the magnitude of regional intrinsic BOLD fluctuations—the relative difference between sexes (FPD > MPD), coupled with the absence of such differences in the healthy PPMI cohort, suggests that the patterns of intrinsic fluctuations in PD are observed differently between the two sex groups. Specifically, while both PD subgroups showed reductions compared to NC, female patients maintained relatively higher fluctuations—a disparity that was not apparent in the healthy cohort. Despite exhibiting more severe visuospatial impairment, female patients maintained relatively higher intrinsic functional oscillations in the bilateral PMC. This sex-divergent pattern suggests that the relationship between regional BOLD fluctuations and cognitive performance may vary between female and male PD patients. However, interpreting this “hyperactivity” as “less efficient brain activity” is overly simplistic. As a non-specific measure of low-frequency BOLD fluctuations (Yang et al., 2018), ALFF does not directly reflect neuronal firing rates or metabolic cost. Rather than being a definitive “pathological basis,” these findings characterize the PMC as a key functional node where the scaling of intrinsic oscillations and clinical deficits diverges between male and female patients. Whether these altered fluctuations represent a distinctive functional reorganization or a direct manifestation of sex-stratified cortical pathology remains to be elucidated, highlighting the complexity of PD-related functional changes beyond a simple linear decline.

Although the results of this study showed sex differences in cortical and subcortical intrinsic regional fluctuations in PD, the underlying mechanisms driving these differences are multifaceted Several factors, including gene expression, environmental factors, and gonadal hormones have been reported to contribute to this dimorphism (Cerri et al., 2019). Gene expression in the SN revealed sex differences in PD (Cantuti-Castelvetri et al., 2007). Males were more exposed to kinds of occupations such as agriculture, metallurgy, and textiles, which were associated with PD incidence (Vlaar et al., 2018). A prior study showed that female PD patients have higher dopamine in the striatum, possibly due to the activity of estrogens and long-term estrogen exposure (Haaxma et al., 2007), and estrogen can enhance synthesis and the release of dopamine, and modify the activity of dopamine neurons (Becker, 1990; Xiao and Becker, 1994). Overall, the interaction between the dysregulation of gene expression, estrogen protective effect, and environmental exposure may results in sex differences in brain activity in PD patients.

A methodological strength of our study is the rigorous control of structural confounders. Since brain morphology has been shown to correlate with intrinsic BOLD signal dynamics (Qing and Gong, 2016), failing to account for structural variation could lead to results that are merely secondary to regional atrophy. By adjusting for GM density and volume, we identified sex-related functional signatures that appear functionally intrinsic. However, we acknowledge the potential concern that such a conservative approach might partition out some shared biological variance inherent to PD pathology. Future studies integrating multimodal imaging are warranted to further disentangle the complex interplay between structural degeneration and functional reorganization in male versus female PD patients.

Several limitations should be considered. First, the sample sizes of male and female PD patients were initially imbalanced; to address this, we conducted a subgroup analysis with matched sample sizes. Furthermore, all reported results were subjected to stringent statistical thresholds, including multiple-comparisons correction as recommended to minimize false-positive findings (Woo et al., 2014), which supports the reliability of the significant outcomes. Second, while our primary NC cohort had a limited number of female participants, we performed a supplementary validation using an independent, relatively sex-balanced NC cohort (*N* = 24, Females/Males = 14/10) sourced from the PPMI database. After controlling for age, education, and gray matter density, no significant clusters survived the stringent GRF correction (voxel *p* < 0.001, cluster *p* < 0.05) across the whole brain. This baseline consistency in a protocol-consistent cohort suggests that the sex-divergent patterns observed in the PD group are likely disease-specific rather than a reflection of innate physiological dimorphism. Nevertheless, we acknowledge that the absence of a formal group sex interaction analysis and the relatively modest scale of the validation cohort necessitate a cautious interpretation regarding the disease-specificity of these findings. Third, the SN’s small volume relative to our resolution (3 × 3 × 3 mmł) and 6 mm smoothing kernel increases risks of partial volume effects and signal spillover. While the refined AAL3 atlas was used, these SN findings remain preliminary and require future high-resolution validation. Then, while ALFF is more susceptible to physiological noise than fractional ALFF (fALFF), our GSR analysis (Supplementary Figure S1) confirmed the spatial stability of these sex-divergent patterns against global artifacts. As a measure of regional dynamics rather than inter-regional coordination, ALFF provides a localized perspective that complements large-scale functional connectivity. Future multi-modal research integrating nodal and edge-based metrics is needed for a holistic view of PD brain organization.

Furthermore, it is important to recognize that ALFF serves as a non-specific measure of low-frequency spontaneous BOLD fluctuations. While it is widely used as a proxy for intrinsic brain activity, it does not directly reflect neuronal firing rates or metabolic efficiency. In a neurodegenerative context, alterations in ALFF can be influenced by multiple factors, including local atrophy, neurovascular coupling changes, and primary pathological processes. Therefore, our interpretations of “higher” or “lower” ALFF should be viewed as regional functional signatures rather than definitive evidence of specific physiological states such as neuroprotection or compensation.

## 5 Conclusion

In conclusion, this study identifies potential sex-divergent patterns in functional BOLD fluctuations in PD, primarily involving higher fluctuations in the bilateral PMC in females, along with a preliminary premotor-visuospatial association. SN alterations remain preliminary. Supplementary analyses in a balanced healthy cohort provide preliminary support suggesting that these alterations are not present at baseline and may be disease-related. Our findings highlight the importance of sex-stratified neuroimaging and provide preliminary support for sex-sensitive functional signatures in PD. However, given the relatively small sample size of the validation cohort and the lack of formal interaction testing, these results should be interpreted as suggestive rather than definitive. Further validation in larger, longitudinal, and demographically balanced studies is required to establish the disease-specificity of these sex-divergent effects.

## Data Availability

The raw data supporting the conclusions of this article will be made available by the authors, without undue reservation, upon reasonable request.

## Footnotes

1
http://www.ppmi-info.org/
