# Supplementary material for: Sex-divergent intrinsic brain function in Parkinson’s disease: elevated nigral fluctuations and premotor-visuospatial coupling in female patients
Source: Front Neurosci. 2026 Jun 15;20:1734858. doi: 10.3389/fnins.2026.1734858 (PMC13310980; doi:10.3389/fnins.2026.1734858)
Supplement: Supplementary file 1 [file Data_Sheet_1.docx]

**Table S1.** Clusters of significant VBM alterations between group comparisons.

| **Anatomical regions** | **BA** | **Cluster size (voxel)** | **MNI (x, y, z)** | ***T* values** |
| --- | --- | --- | --- | --- |
| **NC>FPD** |  |  |  |  |
| NA |  |  |  |  |
| **NC<FPD** |  |  |  |  |
| R Orbitofrontal gyrus | 11 | 156 | 23, 57, -23 | -7.53 |
| L Calcarine | 17/18 | 372 | -5, -107, -11 | -10.07 |
| L Para hippocampal |  | 119 | -23, -23, -12 | -5.25 |
| R Angular | 40 | 104 | 39, -57, 39 | -6.38 |
|  |  |  |  |  |
| **NC>MPD** |  |  |  |  |
| R Middle frontal gyrus | 46 | 184 | 32, 15, 38 | 8.69 |
| L Supramarginal | 48 | 62 | -47, -35, 33 | 6.82 |
| **NC<MPD** |  |  |  |  |
| R Postcentral | 2 | 102 | 45, -36, 54 | -6.25 |
| R Orbitofrontal gyrus | 11 | 170 | 23, 57, -23 | -8.28 |
| L Hippocampus |  | 279 | -23, -21, -12 | -6.88 |
| R Putamen |  | 113 | -6, 0, -6 | -7.21 |
| R Lingual | 18 | 85 | 32, -101, -17 | -6.34 |
| L Calcarine | 17 | 129 | -6, -107, -11 | -8.23 |
| L Fusiform gyrus | 19/37 | 111 | -41, -68, -8 | -6.63 |
|  |  |  |  |  |
| **FPD>MPD** |  |  |  |  |
| NA |  |  |  |  |
| **FPD<MPD** |  |  |  |  |
| Brainstem |  | 717 | 3, -38, -24 | -5.45 |
| R Fusiform gyrus | 19/37 | 413 | 50, -69, -21 | -6.76 |
| L Fusiform gyrus | 19/37 | 276 | -57, -59, -29 | -5.39 |

***Note***: VBM, voxel-based morphometry; NA, not available means no result between the group comparison. BA, Brodmann area; MNI, Montreal Neurological Institute; NC, normal control; FPD, female Parkinson’s disease patients; MPD, male Parkinson’s disease patients; L, left; R, right. The results were thresholded by using a voxel wise threshold *p* < 0.001 and cluster-corrected threshold *p* < 0.05.

**Table S2.** Clusters of significant ALFF alterations between group comparisons.

| **Anatomical regions** | **BA** | **Cluster size (voxel)** | **MNI (x, y, z)** | ***T* values** |
| --- | --- | --- | --- | --- |
| **NC>FPD** |  |  |  |  |
| R Cerebellum |  | 212 | 36, -78, -36 | 5.10 |
| L Cerebellum |  | 113 | -45, -60, -33 | 4.99 |
| L Orbital gyrus | 11 | 14 | -12, 33, -30 | 5.73 |
| L Superior temporal gyrus | 21/22 | 19 | -51, -6, -6 | 8.33 |
| B Frontal/Prefrontal gyrus | 6/8/9/10 | 371 | 24, 39, 36 | 7.27 |
| **NC<FPD** |  |  |  |  |
| R Insula | 13 | 59 | 27, 3, 6 | -6.62 |
| R Putamen |  | 22 | 27, 3, 8 | -6.48 |
| L Insula | 48 | 33 | -30, 27, 3 | 5.23 |
| L Fusiform gyrus | 37 | 35 | -36, -57, -18 | -4.78 |
| B Calcarine | 30 | 74 | -9, -45, 3 | -8.59 |
|  |  |  |  |  |
| **NC>MPD** |  |  |  |  |
| R Cerebellum |  | 90 | 45, -78, -33 | 5.34 |
| L Cerebellum |  | 65 | -51, -66, -36 | 6.69 |
| R Superior temporal gyrus | 21/22/38 | 88 | 33, 9, -27 | 7.55 |
| L Superior temporal gyrus | 21/22/38 | 100 | -51, 15, -15 | 8.49 |
| L Orbital gyrus | 11 | 35 | -15, 27, -27 | 6.21 |
| B Frontal/Prefrontal gyrus | 6/8/9/10 | 705 | -9, 51, 27 | 10.05 |
| L Thalamus |  | 23 | -6, -6, 3 | 7.80 |
| R Cuneus | 18/19 | 84 | 18, -78, 21 | 5.42 |
| L Cuneus | 18 | 63 | -21, -87, 12 | 6.73 |
| **NC<MPD** |  |  |  |  |
| R Insula | 47 | 143 | 51, 21, -3 | -6.84 |
| R Putamen |  | 42 | 29, -1, 8 | -4.98 |
| L Insula | 48 | 128 | -39, -6, 15 | -8.14 |
| L Putamen |  | 27 | 27, 1, 8 | -5.04 |
| R Fusiform gyrus | 37 | 82 | 45, -42, -15 | -5.57 |
| L Fusiform gyrus | 37 | 120 | -39, -60, -18 | -6.09 |
| B Calcarine | 30 | 81 | 12, -48, 6 | -8.15 |
| R Anterior cingulum | 32 | 30 | 12, 36, 24 | -5.96 |
|  |  |  |  |  |
| **FPD>MPD** |  |  |  |  |
| R Premotor cortex | 6 | 38 | 27, 6, 48 | 4.69 |
| L Premotor cortex | 6 | 54 | -27, 6, 51 | 6.55 |
| **FPD<MPD** |  |  |  |  |
| NA |  |  |  |  |

***Note***: ALFF, amplitude of low-frequency fluctuation; NA, not available means no result between the group comparison. BA, Brodmann area; MNI, Montreal Neurological Institute; NC, normal control; FPD, female Parkinson’s disease patients; MPD, male Parkinson’s disease patients; L, left; R, right. The results were thresholded by using a voxel wise threshold *p* < 0.001 and cluster-corrected threshold *p* < 0.05 (GRE-corrected).

**Part I: Robustness Analyses**

- 1. **Demographic-Matched Subset Analysis**

To address the potential impact of sample size imbalance and demographic variances, we performed a secondary analysis using a strictly matched subset (N=58).

- **Table S3:** Demographic and neuropsychological characteristics of the matched subset.
- **Validation Results:** In this matched cohort, the ALFF values in the right SN were significantly higher in females than in males (*p* < 0.013, see Figure 2 in the main text), consistently supporting the trend observed in the total sample. Cortical ALFF alterations (PMC) were also coincident with the primary findings.
  1. **Impact of Global Signal Regression (GSR)**

To evaluate whether our primary findings were influenced by global physiological artifacts, we re-analyzed the data using Global Signal Regression (GSR).

- **Results:** While the primary results in the main text are presented without GSR, Supplementary Figure S1 displays the ALFF patterns with GSR applied. The sex-divergent ALFF signatures remained spatially consistent across both pipelines, confirming that the observed patterns are robust and not driven by global signal fluctuations.

**Table S3.** Demographic and neuropsychological characteristics in sample size matched PD groups.

|  | **Female (*n* = 29)** | **Male (*n* = 29)** | ***P* Value** |
| --- | --- | --- | --- |
| **Age (years)** | 59.62±9.88 | 62.58±11.40 | 0.294 |
| **Education (years)** | 15.62±3.28 | 15.48±2.86 | 0.865 |
| **Disease Duration (months)** | 6.93±9.33 | 6.48±6.64 | 0.834 |
| **MDS-UPDRS III** | 20.69±10.02 | 24.55±12.25 | 0.194 |
| **GDS** | 2.52±2.91 | 2.76±2.16 | 0.721 |

***Note*:** Data are expressed as mean (standard deviation). *P* values were derived from two sample *t*-test for parametric test. GDS = Geriatric Depression Scale; MDS-UPDRS Ⅲ = Movement Disorder Society-Unified Parkinson's Disease Rating Scale Ⅲ.

**Figure S1.** The ALFF alteration of global cortex with global signal regression.


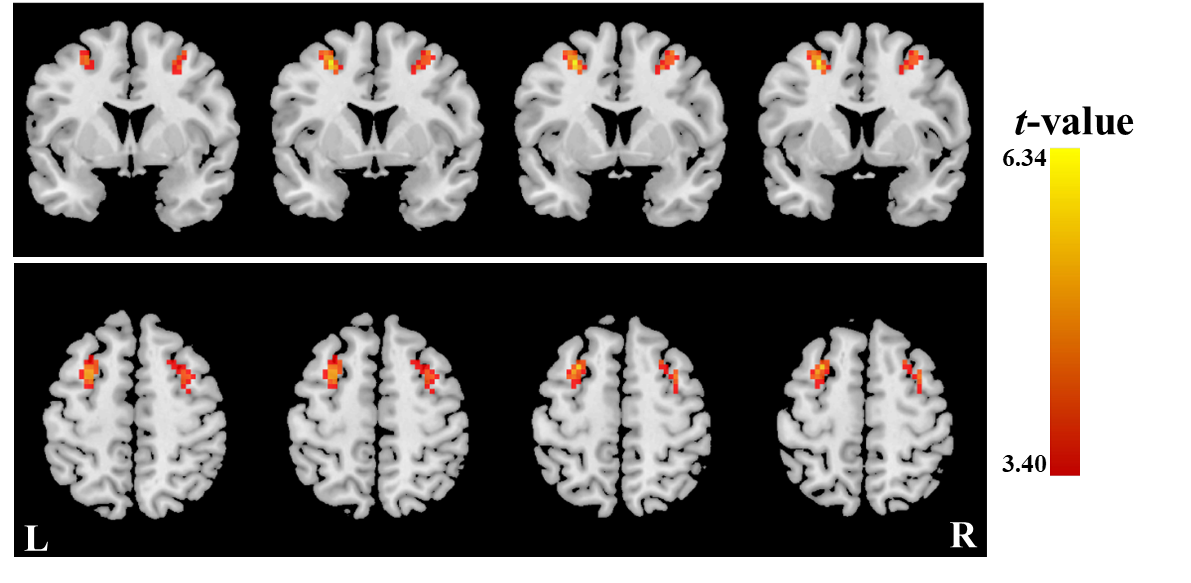


The warm color indicates a higher ALFF value for females than male PD patients when the global signal was entered to covariates in data preprocessing. Color bars indicate t scores. Note: ALFF, amplitude of low-frequency fluctuations; PD, Parkinson’s disease.

**Part II: Supplementary Validation of Disease-Specificity**

**Section S1. Methodology and Results of the Independent NC Validation**

**1. Rationale:**

To ensure that the sex-divergent functional patterns observed in the PD group were not a reflection of innate physiological sexual dimorphism, we conducted a supplementary validation analysis using an independent, relatively sex-balanced healthy control (NC) cohort.

**2. Participants and Methods:**

An independent cohort of 24 healthy controls (14 females and 10 males) was identified from the PPMI database (collected 2021–2024). This cohort was matched for years of education. Demographic variables were analyzed using an independent samples t-test. A voxel-wise whole-brain group comparison (Females vs. Males) was performed using within the PD group using one-way analysis of covariance (ANCOVA) via the DPARSF software. To eliminate potential confounding factors, **age, years of education, and regional grey matter density (GMD)** were included as covariates in the statistical model. Multiple-comparisons correction was applied using the Gaussian Random Field (GRF) method (voxel-level *p* < 0.001, cluster-level *p* < 0.05).

**3. Results:**

Demographic characteristics of this validation cohort are detailed in **Table S4**. After strictly controlling for age,education and structural variations, **no significant clusters survived the GRF correction across the entire brain.** Specifically, no sex differences in ALFF were identified within the target regions (bilateral PMC and right SN) that showed significant alterations in the PD group. This lack of baseline dimorphism supports the notion that the sex-divergent functional patterns reported in our study are disease-specific manifestations of **PD pathology.**

Table S4. Demographic Characteristics of the Supplementary NC Validation Cohort (PPMI)

| Characteristics | NC Females (n=14) | NC Males (n=10) | t | *p* |
| --- | --- | --- | --- | --- |
| Age(years) | 67.21±6.54 | 73.30±5.40 | 2.41 | 0.03 |
| Education (years) | 17.71±2.89 | 19.00±2.11 | 2.19 | 0.25 |

Note: Although a significant difference in age was observed between the validation subgroups (p=0.03), this factor was strictly controlled for as a covariate in the voxel-wise ANCOVA to ensure the independence of the functional results.
